# RNA binding proteins in osteoarthritis

**DOI:** 10.3389/fcell.2022.954376

**Published:** 2022-08-08

**Authors:** Qian Yi, Zhenhan Deng, Jiaji Yue, Jinglong He, Jianyi Xiong, Wei Sun, Weichao Sun

**Affiliations:** ^1^ Department of Bone and Joint Surgery, Shenzhen Second People’s Hospital (The First Affiliated Hospital of Shenzhen University), Shenzhen, China; ^2^ Department of Orthopaedics, Affiliated Hospital of Putian University, Putian, China; ^3^ Department of Physiology, School of Basic Medical Science, Southwest Medical University, Luzhou, China; ^4^ Department of Sports Medicine, The First Affiliated Hospital of Shenzhen University, Shenzhen Second People’s Hospital, Shenzhen, China; ^5^ The Central Laboratory, Shenzhen Second People’s Hospital (The First Affiliated Hospital of Shenzhen University), Shenzhen, China

**Keywords:** RNA binding proteins, osteoarthritis, alternative splicing, mRNA stability, RNA metabolism

## Abstract

Osteoarthritis (OA) is a common chronic degenerative joint disease worldwide. The pathological features of OA are the erosion of articular cartilage, subchondral bone sclerosis, synovitis, and metabolic disorder. Its progression is characterized by aberrant expression of genes involved in inflammation, proliferation, and metabolism of chondrocytes. Effective therapeutic strategies are limited, as mechanisms underlying OA pathophysiology remain unclear. Significant research efforts are ongoing to elucidate the complex molecular mechanisms underlying OA focused on gene transcription. However, posttranscriptional alterations also play significant function in inflammation and metabolic changes related diseases. RNA binding proteins (RBPs) have been recognized as important regulators in posttranscriptional regulation. RBPs regulate RNA subcellular localization, stability, and translational efficiency by binding to their target mRNAs, thereby controlling their protein expression. However, their role in OA is less clear. Identifying RBPs in OA is of great importance to better understand OA pathophysiology and to figure out potential targets for OA treatment. Hence, in this manuscript, we summarize the recent knowledge on the role of dysregulated RBPs in OA and hope it will provide new insight for OA study and targeted treatment.

## Introduction

Cell fate decisions including cellular identity, differentiation, morphology, and phenotype are directed by complex regulatory machinery (Schuschel et al., 2020). The regulatory mechanisms for precise patterns of gene expression range from epigenetic modifications in the transcript level, RNA modifications in posttranscriptional level to posttranslational modifications of the proteome (Zhang and Cao, 2019). The initiation of gene regulation is carried out by proteins that bind specific domain of DNA or RNA molecules. One class of such proteins is transcription factors (TFs), which regulated gene transcription by binding with DNA sequences (Neefjes et al., 2020). Another class of such proteins is RNA-binding proteins (RBPs), which primarily regulated RNA metabolism *via* binding to their target RNAs (Zhao et al., 2017). RBPs-mediated posttranscriptional regulation changes protein expression relatively quickly compared with TF-mediated transcription regulation. These precise regulation machineries contribute to the cellular homeostasis.

Posttranscriptional regulation is one important step of gene expression regulation (Lee et al., 2021). TFs regulated the produce of RNA transcript, and the maturation and fate of the mRNA was regulated by noncoding RNAs (e.g., miRNAs) and RBPs (Bansal and Arora, 2020; Kinoshita et al., 2021). RBPs are a series of proteins which could bind with double or single-stranded RNA and thereby demonstrate the function to influence the RNA fate by forming ribonucleoprotein (Glisovic et al., 2008; Bohnsack and Bohnsack, 2019). The main role of RBPs is RNA metabolism regulation, including mRNA stability (Shi et al., 2017), mRNA splicing (Rachez et al., 2021), translocation (Tolino et al., 2012), translation (Moore and von Lindern, 2018) as well as the corporation with noncoding RNAs (Ciafre and Galardi, 2013; Qin et al., 2020). The functions of RBPs are highly dependent on their structural features which is the RNA binding domains, and the most common domains are including RNA recognition motif (RRM), arginine-glycine-glycine (RGG) motif, the hnRNP-K homology domain (KH), cold-shock domain (CSD), double-stranded RBD (dsRBD), tyrosine rich domain and the zinc finger domain (ZF) (Maris et al., 2005; Dominguez et al., 2018; Qin et al., 2020; Dobrev et al., 2021).

Due to their important roles in gene expression regulation, RBPs are critical in human biology and development. Functional disruption of RBPs, thus, leads to lots of human diseases including cancers, metabolic-related diseases, inflammatory diseases, and so on. Osteoarthritis (OA) is a novel metabolic-related and inflammatory-related disease featured by changed cell morphology and the aberrant gene expression pattern (Martel-Pelletier et al., 2016). Chondrocytes are a single cell type in cartilage tissue that secrete type II collagen and are surrounded by a large number of extracellular matrix proteins. During the OA progression, the morphology and the expression of extracellular matrix proteins of chondrocytes were remarkedly changed (Liu et al., 2016; Aladal et al., 2022). Researchers reported that posttranscriptional alterations play a significant function in OA progression. For example, miR-877-5p alleviates chondrocyte dysfunction in OA models via repressing FOXM1 expression by binding with 3′-UTR of FOXM1 mRNA. Next, targeted METTL3 inhibition could alleviate the senescence of fibroblast-like synoviocytes (FLS) via posttranscriptional regulation of ATG7 and limit OA development in experimental animal models, providing a potential strategy for OA therapy (Chen et al., 2022).

In recent times, Yutaro Uchida and others summarized the emerging roles of RBPs in regulating inflammation diseases, such as rheumatoid arthritis (Uchida et al., 2019). Moreover, researchers reported that the concentrations of cold-inducible RNA-binding protein in synovial fluid are associated with severity in knee OA (Yu et al., 2017). In addition, in Wu’s article, they discussed the therapeutic potential and role of miRNA, lncRNA, and circRNA in OA, and they pointed that RBPs participated in this network (Wu et al., 2019). However, the biological significance of RBPs in OA remains unsystematically discussed. Emerging evidence indicated that lots of RBPs are dysregulated, which influences the development and progression of OA. Therefore, in our recent review, we will briefly summarize the current knowledge on the role of RBPs in OA.

## RNA-binding proteins in regulating RNA metabolism

**FIGURE 1:**
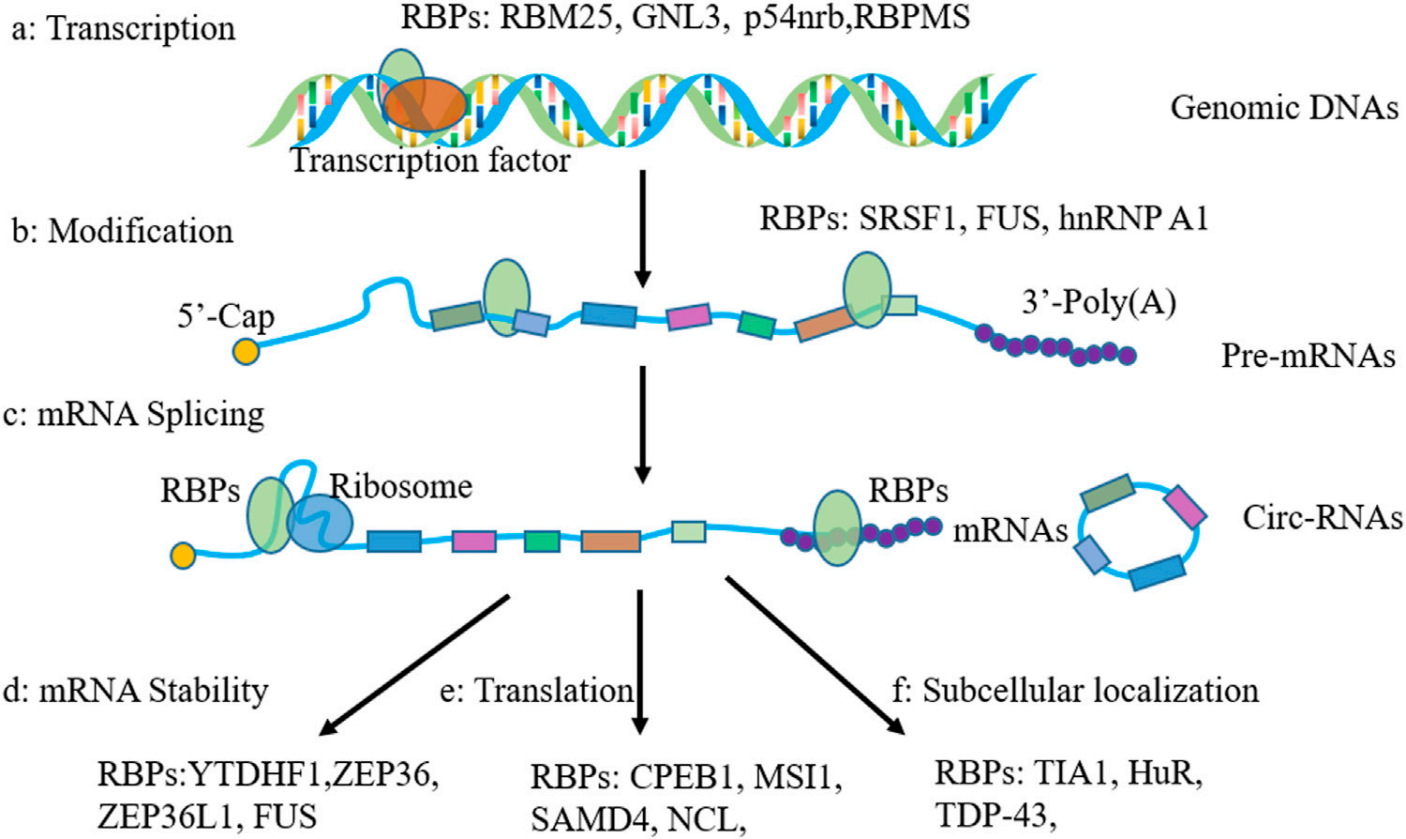
Functions of RNA-binding proteins in regulating gene expression. **(A)** regulation in gene transcription; **(B)** mRNA modifications; **(C)** function in RNA splicing; **(D)** Function in regulating RNA stability; **(E)** regulation of gene translation; **(F)** regulation of mRNA subcellular localization.

RNA metabolism consists of RNA synthesis, folding/unfolding, transport, modifications, processing, translocation as well as degradation. RBPs were reported, which demonstrate an important role in these processes. In this section, we will review the general role of RBPs in RNA metabolism (Figure 1).

### RNA synthesis

First, except traditional transcript factors regulated RNA synthesis, evidence suggests that some RBPs are also demonstrate function in regulating transcription. For example, RBM25 was an RBP involved in splicing regulation, and YY1 was a known RNA-dependent transcription factor. Researchers reported that RBM25 depletion attenuates all YY1-dependent chromatin binding, DNA looping, and transcription (Xiao et al., 2019). Moreover, IGFBP3 participated in the repair of double strand breaks via forming a nuclear complex with EGFR and DNA-PKCs, and then, it promoted the stability of tumor genome in response to DNA-destructive chemotherapy (Lin et al., 2014). Furthermore, researchers reported that nucleolar GTP binding protein 3, GNL3, participates in genomic regulation as RNA-binding protein and upregulates IL24 and PTN expression to promote the development of OA (Zhu et al., 2021).

### Modifications

Second, RBPs also participated in pre-mRNA postprocessing, adding a poly (A) tail at the 3′ end of the mRNA or a cap at the 5′ end (Qin et al., 2020). The presence of methylated guanosyl cap and the poly (A) tail mediate the stability of mRNAs and protects them from exonuclease degradation. The length of poly (A) tail can be influenced by RBPs, and then, the stability, subcellular location, and translation efficiency of target mRNAs were regulated (Darnell, 2013). Researchers illustrated that cytosolic polyadenylation element-binding protein (CPEB1) extended poly (A) tails of lots cell cycle-related mRNAs and enhanced their translation efficiency (D'Ambrogio et al., 2013). Moreover, CPEB1 can directly target the 3′-UTR of SIRT1, control the poly (A) tail length and inhibit its translation, and suppress the stemness and resistance of liver cancer (Xu et al., 2018). In addition, RBPs is also involved in the m6A modification of mRNAs by regulating methyltransferase installation. Researchers reported that RBM15 and Zc3h13 associated with Mettl3, Mettl14, and WTAP to form methyltransferase complex (Wen et al., 2018).

### Alternative splicing

Third, RBPs regulated RNA splicing, another major pre-mRNA processing event. Some RBPs can interact with core proteins to form spliceosome complexes (Fredericks et al., 2015). The main types of aberrant splicing are constitutive splicing, including exon skipped, exon included, alternative five or three splice-sites, intron retention, and mutually exclusive exon, and they contribute to protein diversity and mRNA stability (Frankiw et al., 2019). For example, RNA-binding protein RBM10 was reported to inhibit the splicing of exon nine of NUMB mRNA (Collins et al., 2017). Feng et al. reported that SRSF1 was responsible for the mechanical stress-induced alternative splicing of cyclin D1 (Feng et al., 2021). RBFOX2 interacted with hnRNPC, hnRNPM, and SRSF1 to regulate splicing of a large number of transcripts implicated in cell differentiation and development (Zhou et al., 2021). In recent times, RBPs have also been reported to participate in the splicing and formation of circRNAs. Ni and others reported that FUS binds to the GUGGU sequences at nt 358 and 409 proximal to the back-splicing sites (exon 8) and one sequence at nt 499 proximal to the back-splicing sites (exon 10) of SLC7A2 to induce circSLC7A2 formation (Ni et al., 2021).

### Subcellular localization

mRNA was generated in the nucleus and could be translocated into the cytoplasm. RBPs can recognize the cis motif or secondary structure of mRNA and mediate the localization of RNA to a specific subcellular compartment. The hnRNPs family member proteins are in charge of RNA transporting from the nucleus into the cytoplasm (Mofatteh and Bullock, 2017). More importantly, a recent study also revealed that RBPs mediated the translocation of mRNAs to stress granule (SGs), a ribonucleoprotein complex. Researchers reported that TIA-1 and HuR bind selectively to mRNAs with high AREs in their UTRs and recruit them to SGs (Bley et al., 2015). What’s more, the RBP TIA-1 binds p53 mRNA and mediates it with RNA particle localization. When the cell was damaged, p53 mRNA is released from the stress particles and binds to polysomes, resulting in the translation of p53 (Diaz-Munoz et al., 2017). Poly (A) binding protein (PABP), a cytoplasmic RNA-binding protein, regulated mRNA translation and stability by binding to the 3′ poly (A) tail. Researchers reported that PABP recruited localized mRNA and translation at the cell membrane (Bohm et al., 2018). In addition, recently studies reported that some RBPs showed significant function in regulating mRNA sorting to exosomes. Ann L Wozniak and others revealed that the RNA-binding protein FMR1 controls selective exosomal miRNA cargo loading during inflammation (Wozniak et al., 2020). Also, they reported that sumoylated hnRNPA2B1 controls the sorting of miRNAs into exosomes through binding to specific motifs (Villarroya-Beltri et al., 2013).

### RNA stability

The eukaryotic mRNAs will be degradation. The cap structure at the 5′ end and poly (A) at the 3′ end play an important function in preventing mRNAs degradation (Hamilton et al., 2010). Researchers reported that almost 16% of transcripts 3′-UTR contain an AU-rich original, which is the main structure involved in reversing degradation of mRNAs (Gruber et al., 2011). Also, in this process, AU-rich original binding proteins promote the mRNA degradation or enhance their stability (Fallmann et al., 2016). Researchers reported that PTBP3 binds to the 3′-UTR of ZEB1 mRNA to prevent its degradation and promote EMT of breast cancer (Hou et al., 2018). Moreover, our group reported that CIRP binds to the 3′-UTR of CTNNB1 mRNA to promote its stability and the progression of nonsmall lung cancer (Liao et al., 2021). Furthermore, ZFC3H, RBP zinc-finger C3H1 domain-containing protein, play a crucial role in the regulating the degradation of nuclear RNAs *via* acting as an adaptor protein mediated the interaction between nuclear RNAs and the exonucleases complex (Silla et al., 2018). Next, the insulin-like growth factor 2 mRNA-binding proteins (IGF2BPs) are a family of well-conserved mRNA-binding proteins consisting of two RRMs and four KH domains, and their cellular function is stabilization of bound mRNAs (Degrauwe et al., 2016). In addition, researchers reported that YTHDF1 mediated the stability of Bcl2 mRNA and inhibited the apoptosis and autophagy of chondrocytes in inflammation (He et al., 2022a).

### Translation

The translation process is complex and exhibits an initiation stage, extension stage, and termination stage. Studies showed that RBPs eIF4F and PABP are involved in the translation activation. An internal ribosome entry site (IRES) is found in the 5′-UTR sequence of some mRNAs. Our group reported that CIRP binds with IRES sequence of CTNNB1 mRNA and promotes its translation (Liao et al., 2021). In addition to binding with the 5′-UTR of mRNAs to influence translation, some RBPs regulate the translation by binding 3′-UTR. For example, researchers reported that MSI1 binds to the 3′-UTR untranslated regions of mRNAs, interacts with the poly (A)–binding protein, and competes for eIF-4G. As a result, it disturbs the translation initiation (Kawahara et al., 2008). Further, MSI2 was reported promoted tumorigenesis by the inhibition of NUMB mRNA translation (Pereira et al., 2012). Moreover, Dab2 and ILEI mRNAs 3′-UTR contain TGF-β-activated translation (BAT) stem-loop elements, which can be recognized by hnRNPE1, and then, the translation was silenced (Chaudhury et al., 2010). Also, interestingly, hnRNP-K could enhance the translation of c-myc mRNA by binding to the internal ribosome entry site in the 5′-UTR (Evans et al., 2003), and it also could silence the translation of 15 lipoxygenase (LOX) mRNA by binding to its differentiation control element located in the 3′-UTR (Ostareck et al., 1997).

## RNA-binding proteins in osteoarthritis

**FIGURE 2:**
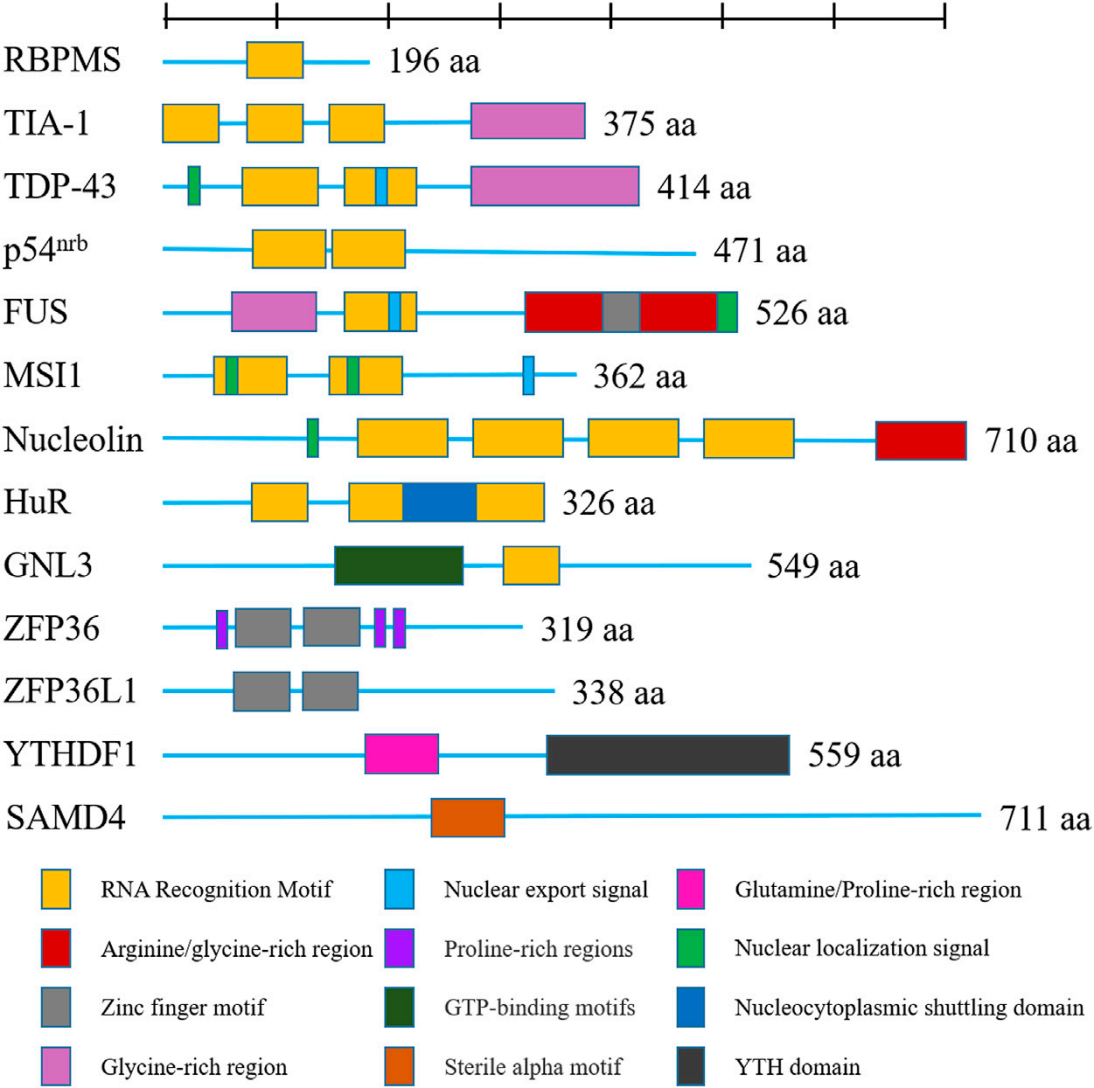
Shown are human RNA-binding proteins playing a major role in osteoarthritis as discussed in this review. Domains are color-coded and proteins are scaled to amino acid length. Different colors represent different functional domains.

RBPs are increasingly revealed as crucial players in OA, as they have been proven to play an important function in the regulation of gene expression by influencing RNA metabolism. In the past decade, mounting studies illustrated that the role of RBPs in OA *via* their actions in RNA metabolism, including transcription, RNA alternative splicing, and mRNA stability. In our present review, we will discuss the function of RBPs in OA in the following aspects: 1) regulation in gene transcription, 2) function in RNA splicing, 3) function in regulating RNA stability, 4) RBPs in stress granules, 5) RBPs in regulating translation. The RBPs playing a major role in OA discussed in this review were shown in Figure 2.

### RNA-binding proteins in regulating gene transcription in osteoarthritis

As we all know, the gene transcription regulation is traditionally known to be mediated by DNA-binding TFs. Recent studies indicate that some RBPs also demonstrate an important function in controlling gene transcription. Splicing factor proline and glutamine-rich protein, SFPQ, has been reported to cooperate with HDAC1 to suppress the CD40 transcription (Pan et al., 2019). Hata et al. illustrated that paraspeckle regulatory protein 54-kDa nuclear RNA-binding protein, p54nrb, associated with SOX9 to enhance the gene promoter activity of Col2a1 and promoted chondrocyte differentiation. Moreover, researchers reported that p54nrb could control the splicing and maturation of Col2a1 mRNA because its RRMs are highly homologous to the RRM domain of polypyrimidine tract-binding protein-associated splicing factor (Hata et al., 2008). Zhu and others reported that nucleolar GTP binding protein 3, GNL3, participates in genomic regulation as RNA-binding protein, and then, it upregulates IL24 and PTN expression to promote the development of OA (Zhu et al., 2021). Furthermore, RNA-binding protein with multiple splicing, RBPMS, has been shown to physically interact with Smads and enhance Smads’ transcriptional activity (Sun et al., 2006). Besides, Shanmugaapriya et al. reported that RBPMS expression was significantly decreased in aged and osteoarthritic cartilage, which suggested its potential role in the maintenance of normal articular cartilage (Shanm ugaapriya et al., 2016). Furthermore, Gu et al. reported that FUS might work together with Runx2 to mediate the transcription of Col10a1 and then regulate chondrocyte hypertrophic differentiation (Gu et al., 2014). In addition, DiGeorge syndrome critical region 8, DGCR8, a double-stranded RNA-binding protein, was reported to play a significant function in maintaining heterochromatin organization and attenuating senescence and OA (Deng et al., 2019).

### RNA-binding proteins in regulating mRNA splicing in osteoarthritis

In recent times, Katsoula et al. performed deep RNA sequencing and genome-wide differential splicing analysis of cartilage tissues of 124 OA patients, and they reported differential splicing for 209 genes, which consists of extracellular matrix proteins, proteoglycans, and integrin surface interactions terms (Katsoula et al., 2022). For example, periostin (POSTN) is a secretory matricellular matrix protein. Researchers reported that splicing isoforms one of POSTN were highly expressed only in chondrocytes and reported to possibly influence cell adhesion (Cai et al., 2019). These findings highlight the important role of gene alternative splicing in OA progression. FUS is an RNA-binding protein that regulates RNA alternative splicing, transcription, and RNA transportation. FUS demonstrates a Gly-rich domain, a RRM domain, two arginine/glycine-rich domain, and a ZF motif. Studies showed that FUS can bind to RNAs that contain enriched GUGGU or GUU motif (Lagier-Tourenne et al., 2012; Zhao et al., 2018). Researchers reported that FUS regulated the mRNA splicing. Ni and others illustrated that FUS binds to the GUGGU sequences at nt 358 and 409 proximal to the back-splicing sites (exon 8) and one sequence at nt 499 proximal to the back-splicing sites (exon 10) of SLC7A2 to promotes circSLC7A2 production. Also, the formed circSLC7A2 protects against OA *via* inhibiting miR-4498/TIMP3 axis (Ni et al., 2021). Moreover, Shen et al. also reported that FUS promoted the splicing of PDE4B pre-mRNA to produce the circPDE4B. Also, they found that circPDE4B was downregulated in OA, and it prevented articular cartilage degeneration and promoted repair through acting as a scaffold for RIC8A and MID1 (Shen et al., 2021). In addition, RBP QKI belongs to STAR family, and it is a splicing factor. Wu et al. reported that QKI promoted the formation of circPDE4D, which inhibited chondrocyte matrix degradation by regulating miR-103a-3p/FGF18 axis (Wu et al., 2021).

### RNA-binding proteins in regulating mRNA stability in osteoarthritis

N6-methyladenosine (m6A) is the most prevalent modification in eukaryotic messenger RNAs (mRNAs). It was installed by “writers”: the m6A methyltransferases including METTL3/14, WTAP, and RBM15/15B, reverted by “erasers,” and the demethylases including FTO and ALKBH5 and recognized by its “readers,” including RBPs YTHDF1/2/3 and IGF2BPs (Huang et al., 2018; Chen et al., 2019). The readers were illustrated to play an important role in regulating the stability of m6A-bearing transcripts. For example, METTL3 facilitates tumorigenesis by enhancing c-Myc mRNA stability via YTHDF1-mediated m6A modification (Zhao et al., 2020). Researchers reported that YTHDF1 mediated the stability of Bcl2 mRNA and inhibited the apoptosis and autophagy of chondrocytes in inflammation (He et al., 2022a). Moreover, Chen et al. reported that METTL3 regulated m6A modification and mRNA stability of ATG7, and then, it affected autophagy to promote cellular senescence and OA progression (Chen et al., 2022). In addition, METTL3 expression was significantly upregulated in degenerative human endplate cartilage tissue. It mediated the methylation of SOX9 mRNA and disrupted its stability, thereby inhibiting the expression of collagen 2 (Xiao et al., 2022).

ZFP36 is an ancient RNA-binding protein that is also known as tristetraprolin (TTP) and TPA-inducible sequence 11 (TIS11). The ZFP36 family consists of ZFP36, ZFP36L1, ZFP36L2, and ZFP36L3. They share the similar structure, including two tandemly repeated ZF motifs (CCCH), which bind to AU-rich elements in the 3′-UTRs of target mRNAs to trigger mRNA destabilization and decay (Bermudez et al., 2011; Sanduja et al., 2011). Researchers reported that ZFP36 binds to AREs in the 3′-UTR of SOX-9 mRNA, acts as a suppressor of SOX-9, and then regulates anabolic and catabolic gene expression in chondrocytes (McDermott et al., 2016). Moreover, Son et al. found that RNA-binding protein, ZFP36L1, was increased in expression in OA chondrocyte. ZFP36L1 bound to the 3′-UTR of the HSPA1A mRNA, enhanced HSPA1A mRNA decay, and reduced the protein level of HSPA1A, which was reported to protect against OA pathogenesis by inhibiting chondrocyte apoptosis (Son et al., 2019).

FUS also has been reported to play function in maintain mRNA stability. For example, FUS binds GluA1 mRNA in the vicinity of the 3′ terminus and controls poly (A) tail maintenance, thus regulating stability (Udagawa et al., 2015). Next, Bai et al. found that FUS worked together with LncRNA-MM2P to bind with and stabilize SOX-9 mRNA and then promoted the chondrocyte-specific protein expression (Bai et al., 2020). HuR demonstrates three RRMs domain and increased the stability of target mRNAs by binding with the AU-rich elements (AREs) of them (Pabis et al., 2019). The 3′-UTR of COX-2 mRNA contains an ARE element, and Riina et al. reported that aurothiomalate inhibited HuR expression and then promoted the destabilization of COX-2 mRNA in human cartilage chondrocytes (Nieminen et al., 2008). In addition, Lv and others reported that the RNA-binding protein SND1 bound with HSPA5 at the 3′UTR, destabilized and suppressed its expression, and then promoted the degradation of GPX4 and ferroptosis in osteoarthritis chondrocytes (Lv et al., 2022).

### RNA-binding proteins in stress granules in osteoarthritis

When cells undergo physiological stress, they will form SGs to respond adequately to stress and adapt to them. SGs are ribonucleoprotein complexes consisting of translationally stalled mRNAs and RBPs, such as TIA-1 and HuR (Wolozin and Ivanov, 2019). The protein structure of TIA-1 contains three RRMs domains and a C-terminal Gly-rich domain, which contribute to stress granule assembly (Zhao et al., 2018). HuR also demonstrates three RRMs domains and prevents the degradation and increase the stability of target mRNAs by binding with the AREs of them (Pabis et al., 2019). Researchers reported that they bind selectively to mRNAs with high AREs in their UTRs and recruit them to SGs (Bley et al., 2015). In IL-1β-treated in OA chondrocytes, TAI-1 and HuR work together to sequester COX-2 mRNAs, which are in SGs, and suppress its expression (Ansari and Haqqi, 2016). What’s more, Liu et al. reported that the expression of HuR was significantly increased in rheumatoid arthritis (RA) synovial tissue, and TNF-α could induce the nucleocytoplasmic shuttling of HuR (Liu et al., 2019). Another important RBP, TDP-43, plays an important role in splicing. It exhibits two RRMs, a nuclear localization signal (NLS), a nuclear export signal, and a Gly-rich domain. It is usually localized in the nucleus, while localized to SGs upon cellular stress (Khalfallah et al., 2018). Chang et al. reported that TDP-43 maintains chondrocyte homeostasis and prevents cartilage degradation in OA (Chang et al., 2021). Moreover, Huang and others revealed that TDP-43 increased RACK1 expression and then promoted SGs formation and inhibited inflammatory response in OA (Huang et al., 2017). Besides, another group also reported the function of TDP-43 in regulating SGs formation, RACK1 expression, inflammatory factors secretion by inhibiting the JNK, and p38 MAPK signaling pathway in OA (Huang et al., 2019).

### RNA-binding proteins in regulating translation in osteoarthritis

CPEB1 (cytoplasmic polyadenylation element-binding protein 1) is an RNA-binding protein that binds the 3′-UTR-cytoplasmic polyadenylation element of its target mRNAs, mediates the extension or removal of poly (A) tail, and then regulates their translation (Udagawa et al., 2013). Researchers reported that CPEB1 was upregulated in articular cartilage from OA patients, and its expression level was correlated with disease severity (Li et al., 2019). MSI1 binds to the 3′-UTR untranslated regions of mRNAs, interacts with the poly (A)-binding protein, and competes for eIF-4G. As a result, it disturbs the translation initiation. Padial-Molina et al. revealed that MSI1 expressed in mesenchymal stromal cells, osteoblasts, and osteocytes, but not in chondrocytes, suggested its different function in the bone healing environment (Kawahara et al., 2008). Next, RNA-binding protein sterile alpha motif domain-containing protein 4 (SAMD4) is a special RNA-binding protein. It contains a sterile alpha motif, which directly binds RNA with stem-loop structures and led the translational repression of target mRNA (Baez and Boccaccio, 2005). Niu et al. found that Samd4-deficient mice displayed chondrocyte defects proved its crucial role in metabolic bone diseases (Niu et al., 2017). Nucleolin (NCL), a type of multifunctional nonribosomal protein, which exhibits four RRM-domains, is mainly found in the nucleolus and plays key roles in ribosome biogenesis. Also, it has been reported to bind with 5′-UTR of p53 mRNA or 3′-UTR of Bcl2 mRNA, respectively, to influence the translation or mRNA stabilization, respectively, (Takagi et al., 2005; Ishimaru et al., 2010). Deng et al. reported that NCL promotes articular chondrocyte proliferation *via* the MAPK/Erk1/2 pathway (Deng et al., 2020). In addition, Lee et al. reported that PUM1 interacted with the 3′-UTR of TLR4 to suppress its mRNA translation, and PUM1 overexpression protected the chondrocytes from inflammation-mediated disruption of the chondrogenic phenotype (Yoon et al., 2022).

## RNA-binding proteins in signaling pathway of osteoarthritis

**FIGURE 3:**
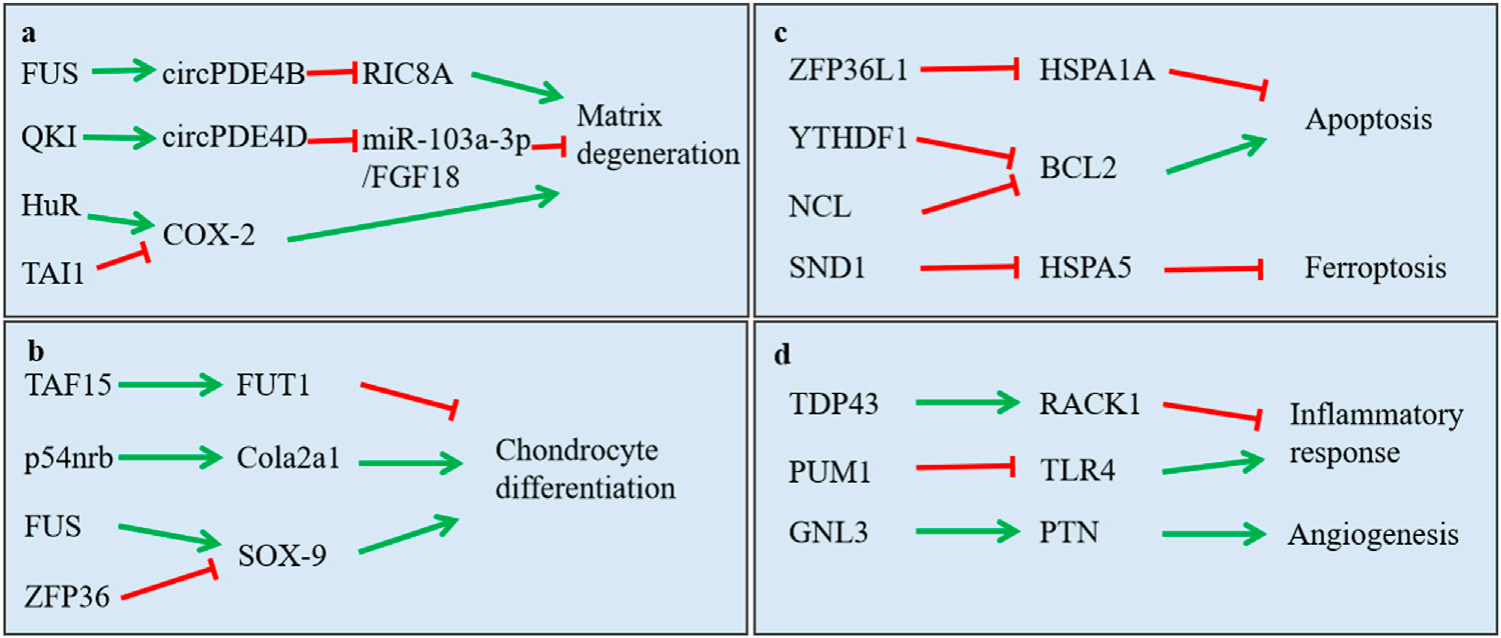
Function of RNA-binding proteins in regulating main signaling pathway in osteoarthritis. **(A)** regulation in extracellular matrix degradation of chondrocytes; **(B)** regulation in chondrocyte differentiation; **(C)** regulation in cell apoptosis, proliferation and ferroptosis; **(D)** regulation in inflammatory response and angiogenesis.

As we all know, OA acted as degenerative disease that was characterized by osteophyte formation, inflammatory response, angiogenesis and aberrant anabolic, and catabolic metabolism-induced cartilage destruction. Growing evidence indicated that RBPs participated in these pathways to effect OA progression, especially anabolic and catabolic metabolism. We summarized the function of RBPs on these pathways in Table 1 and Figure 3.

**TABLE 1:** General overview of osteoarthritis-related RNA-binding proteins and their function.

RBP	Target DNA or mRNA(s)	Function	Phenotype/Function	References
p54nrb	Col2a1	Transcription	Enhance the gene promoter activity of Col2a1 and promoted chondrocyte differentiation	Hata et al. (2008)
GNL3	IL24 and PTN	Genomic regulation	Induce articular osteocyte apoptosis and angiogenesis	Zhu et al. (2021)
RBPMS	Physically interact with Smads	Transcription	Form a counter-regulatory mechanism with TGF-β and IL-1β to maintenance the homeostasis of normal articular cartilage	(Sun et al., 2006) (Shanm ugaapriya et al., 2016)
FUS	Col10a1 promoter	Transcription	Mediate the transcription of Col10a1 and regulate chondrocyte hypertrophic differentiation	Gu et al. (2014)
DGCR8	—	Maintaining heterochromatin organization	Alleviated hMSC aging and osteoarthritis	Deng et al. (2019)
FUS	SLC7A2 mRNA	Splicing	Mediate the formation of circSLC7A2 and inhibit the miR-4498/TIMP3 axis and inflammatory response	Ni et al. (2021)
FUS	PDE4B mRNA	Splicing	Mediate the formation of circPDE4B and regulates chondrocyte cell viability and extracellular matrix metabolism	Shen et al. (2021)
YTHDF1	Bcl2 mRNA	Stability	Inhibited the apoptosis and autophagy of chondrocytes	He et al. (2022a)
METTL3	Atg7 mRNA	Stability	Affected autophagy to promote cellular senescence	Chen et al. (2022)
METTL3	Sox9 mRNA	Stability	Mediated the methylation and degradation of SOX9 mRNA, suppressed collagen 2 expression	Xiao et al. (2022)
ZFP36	Sox9 mRNA	Stability	Increased sox9 expression and then regulating anabolic and catabolic gene expression	McDermott et al. (2016)
ZFP36L1	HSPA1A mRNA	Stability	Enhanced HSPA1A mRNA decay, and inhibited chondrocyte apoptosis	Son et al. (2019)
FUS	Sox9 mRNA	Stability	Stabilized SOX-9 mRNA, and promoted the chondrocyte-specific protein expression	Bai et al. (2020)
HuR	Cox-2 mRNA	Stability	Stabilized cox-2 mRNA and OA progression	Nieminen et al. (2008)
TAI-1	Cox-2 mRNA	Location	Sequester COX-2 mRNA to SGs and delayed its translation	Ansari and Haqqi, (2016)
TDP43	RACK1 mRNA	Location	Promoted SGs formation and inhibited inflammatory response	(Huang et al., 2017; Chang et al., 2021)
CPEB1	—	Translation	CPEB1 overexpression aggravated the catabolic effect of IL-1β on chondrocytes *in vitro*	Li et al. (2019)
SAMD4	Mig6	Translation	Samd4-deficient mice displayed chondrocyte defects	Niu et al. (2017)
Nucleolin	p53 mRNA	Translation	Promotes articular chondrocyte proliferation	(Takagi et al., 2005; Deng et al., 2020)

First, RBPs play important role in anabolic and catabolic metabolism of chondrocytes. Such as SOX-9 being a well-known important factor for chondrocyte differentiation, researchers reported that ZFP36 and FUS both could bind with SOX-9 mRNA to repress or increase it expression, respectively (McDermott et al., 2016) (Bai et al., 2020). They affected the anabolic and catabolic process of chondrocytes by the regulation of SOX-9 expression. Moreover, Chang et al. reported that TDP-43 alleviated cartilage catabolic metabolism and maintained chondrocyte homeostasis under oxidative stress through regulating SGs dynamics (Chang et al., 2021). Furthermore, Shen et al. reported that FUS promoted the formation of circPDE4B. circPDE4B downregulated the expression of catabolic genes including MMP3, MMP13, and ADAMTS4, while it upregulated the expression of anabolic genes, including SOX9 and COL2A1 (Shen et al., 2021). Wu’s group reported that QKI modulated the splicing of PDE4D to form circPDE4D, which protected against OA by inducing the expression of aggrecan and reducing the expression of catabolic enzymes (MMP3, MMP13, ADAMTS4, and ADAMTS5) (Wu et al., 2021).

Second, RBPs also regulated the cell senescence, autophagy, apoptosis, and inflammatory response of mesenchymal stem cells, chondrocytes, and synovial fibroblasts. Researchers reported that PUM1 binds with TLR4 mRNA and suppressed its expression, and then, it inhibited cellular aging of MSCs and OA progression (Yoon et al., 2022). Fu and others reported that TAF15 was upregulated in cartilage tissues from osteoarthritis patient. TAF15 worked with lncRNA XIST to stabilize FUT1 expression and inhibit chondrogenic differentiation of BMSCs (He et al., 2022b). Moreover, researchers reported that DGCR8 could reverse premature senescent phenotypes of hMSCs and prevent and osteoarthritis progression (Deng et al., 2019). Meulenbelt et al. revealed that IGFBP5 and IGFBP6 were involved in the cellular senescence of chondrocytes in osteoarthritis (Houtman et al., 2021). Also, Wang et al. found that YTHDF1 mediated the stability of Bcl2 mRNA and overexpressed BCL2 protein to bind with Beclin1 protein to inhibited autophagy (He et al., 2022a). Furthermore, researchers found that CPEB1 induced chondrocytes apoptosis and promoted joint fracture-induced osteoarthritis (Chen et al., 2020). ZFP36L1 inhibited chondrocytes apoptosis by binding to the mRNA of HSPA1A and increasing its expression (Son et al., 2019). Besides, researchers reported that HuR was related to the chondrocyte hypertrophic differentiation (Gu et al., 2014). In addition, researchers found that HuR expression was significantly elevated in RA FLS, and its expression demonstrates a positive relationship with the NLRP3 inflammasome activation (Liu et al., 2019). Liu et al. reported that the proliferation, migration, and invasion of FLS was related to the expression of PTBP1 (Liu et al., 2021). These results indicated the potential function of RBPs on inflammatory response of OA.

## Discussion and future perspectives

Osteoarthritis is one of the most common joint diseases worldwide, with an incidence of about 28% in the world population over 60 years old. The pathological features of OA are the erosion of articular cartilage, subchondral bone sclerosis, synovitis, and metabolic disorder (Martel-Pelletier et al., 2016; Mandl, 2019). Although various studies showed that gender, age, and obesity are the main risks factors of OA, the pathophysiology of OA was not fully illustrated (Bijlsma et al., 2011; Glyn-Jones et al., 2015). An increasing number of studies showed that the cell morphology is changed, and the aberrant expression of genes involved in inflammation, proliferation, and metabolism are existed in the initiation and progression of OA (Charlier et al., 2019; Li et al., 2021). Significant research efforts are ongoing to elucidate the complex molecular mechanisms underlying OA focused on gene transcription (Fisch et al., 2018). For example, Sox9, an essential transcription factor for cartilage differentiation and function, regulated the transcription of miR-455 to repress HIF-2α expression and coordinately regulate cartilage homeostasis (Ito et al., 2021). Also, importantly, posttranscriptional alterations play significant function in inflammation and metabolic changes related diseases, including OA.

Emerging *in vitro* and *in vivo* research indicated that lots of RBPs are dysregulated in chondrocytes, synovial fibroblasts, and osteoblasts and results in significant effects on OA progress. RNA alternative splicing, RNA stability, transcription, and translation are the major types of RBPs-RNA interaction events contributing to OA development based on acknowledges to date. These studies highlight the potential possibility of targeting RBPs to restore the cellular homeostasis of chondrocytes and prevent the progression of OA. In recent times, RNA interference-based silencing, genetic ablation, or adenovirus-mediated overexpression of RBPs have been used to test their influences on OA progression. For example, Young-Ok Son reported that genetic ablation or silencing of Zfp36l1 significantly abrogates experimental OA in mice (Son et al., 2019). Furthermore, Samd4-deficient mice also display chondrocyte defects and indicated its important function in maintaining chondrocytes homeostasis (Niu et al., 2017). Moreover, Chang and others reported that intraarticular injection of recombinant TDP-43 could significantly prevent ECM degradation of cartilage and subchondral bone remodeling *in vivo* (Chang et al., 2021).

On the other hand, small molecule inhibitors targeting RBPs are being explored. These RBPs-targeting molecules were well used for the treatment of cancers and other diseases. For instance, CMLD-2 and MS-444 are two HuR inhibitors, and their application for colorectal and colon cancer treatment was in the preclinical stage (Wu et al., 2015; Lang et al., 2017). Moreover, Resveratrol was reported an inhibitor of RBFox2, and the clinical trials of it to treat melanoma are now in phase II (Choi et al., 2019). The bioactive plant diterpene oridonin was a direct inhibitor of NCL and has been shown as a special function for using in the treatment of cancer, inflammation, and viral infection (Vasaturo et al., 2018). Further, importantly, researchers reported that aurothiomalate could reduce the expression of HuR and then inhibited the COX-2 expression in chondrocytes and prevented the progression of OA (Nieminen et al., 2008). I-BET151was reported to target and repress the activity of IGF2BP3, and studies showed that I-BET151 suppresses the expression of inflammatory genes and matrix-degrading enzymes in RA synovial fibroblasts (Klein et al., 2016), in addition to suppressing the IL-1β- and TNF-α-induced expression and activity of several matrix-degrading enzymes in human chondrocytes (Dai et al., 2018). Further studies are needed to elucidate the effects and safety of accurate RBPs molecular inhibitor for the OA treatment.

## Conclusion

In summary, this review underlines the function and influence of RBPs in OA and thus the importance of understanding their interaction networks. However, the regulatory network of RBPs in OA is still complex and not completely understood. Future studies will further contribute to this knowledge and will hopefully enable the discovery of novel potential targeted therapies.

## Abbreviations

AREs, AU-rich elements; BAT, TGF-β-activated translation; CPEB1, cytosolic polyadenylation element-binding protein; eIF4, eukaryotic translation initiation factor 4; GNL3, nucleolar GTP binding protein three; HuR, human antigen R; IGFBP3, insulin-like growth factor binding protein three; IRES, internal ribosome entry site; lncRNA, long noncoding RNA; MSI1, Musashi1; ncRNAs, noncoding RNAs; NCL, nucleolin; OA, osteoarthritis; PABP, poly (A) binding protein; RBPs, RNA binding proteins; RBM, RNA-binding motif; RRMs, RNA recognition elements; RGG, arginine/glycine-rich region; SR, serine/arginine; SGs, stress granules; SFPQ, splicing factor proline and glutamine-rich protein; TEAD, TEA domain transcriptional; 3′-UTR, 3′-untranslated regions; ZEB1, zinc-finger E-box binding homeobox one; ZFP36, zinc-finger protein homolog 36; Znf, zinc-finger motif
